# Melatonin can be, more effective than N-acetylcysteine, protecting acute lung injury induced by intestinal ischemia-reperfusion in rat model

**DOI:** 10.6061/clinics/2021/e2513

**Published:** 2021-04-26

**Authors:** Alberto Andrade Leite, Russel Joseph Reiter, Julio Cezar Mendes Brandão, Thiago Mamoru Sakae, Marcia Marinho, Celia Regina Camargo, Itamar Souza Oliveira-Junior

**Affiliations:** I Programa de Pos-Graduacao em Medicina Translacional, Universidade Federal de Sao Paulo, Sao Paulo, SP, BR; II Department of Cell Systems and Anatomy, UT Health Science Center at San Antonio, San Antonio, Texas, USA; III Departamento de Cirurgia, Disciplina de Anestesiologia, Dor e Medicina Paliativa, Universidade Federal de Sergipe, Aracaju, SE, BR; IV Universidade do Sul de Santa Catarina, Santa Catarina, SC, BR; V Departamento de Producao e Saude Animal, Universidade Estadual Paulista, Faculdade de Medicina Veterinaria, Aracatuba, SP, BR; VI Departamento de Cirurgia, Disciplina de Anestesiologia, Dor e Medicina Intensiva, Universidade Federal de Sao Paulo, Sao Paulo, SP, BR

**Keywords:** Melatonin, Intestinal Ischemia-Reperfusion, NAC, Acute Respiratory Distress Syndrome, Experimental Model

## Abstract

**OBJECTIVES:**

The current study compared the impact of pretreatment with melatonin and N-acetylcysteine (NAC) on the prevention of rat lung damage following intestinal ischemia-reperfusion (iIR).

**METHODS:**

Twenty-eight Wistar rats were subjected to intestinal ischemia induced by a 60 min occlusion of the superior mesenteric artery, followed by reperfusion for 120 min. Animals were divided into the following groups (n=7 per group): sham, only abdominal incision; SS+iIR, pretreated with saline solution and iIR; NAC+iIR, pretreated with NAC (20 mg/kg) and iIR; MEL+iIR, pretreated with melatonin (20 mg/kg) and iIR. Oxidative stress and inflammatory mediators were measured and histological analyses were performed in the lung tissues.

**RESULTS:**

Data showed a reduction in malondialdehyde (MDA), myeloperoxidase (MPO), and TNF-alpha in the animals pretreated with NAC or MEL when compared to those treated with SS+iIR (*p*<0.05). An increase in superoxide dismutase (SOD) levels in the NAC- and MEL-pretreated animals as compared to the SS+iIR group (34±8 U/g of tissue; *p*<0.05) was also observed. TNF-α levels were lower in the MEL+iIR group (91±5 pg/mL) than in the NAC+iIR group (101±6 pg/mL). Histological analysis demonstrated a higher lung lesion score in the SS+iIR group than in the pretreated groups.

**CONCLUSION:**

Both agents individually provided tissue protective effect against intestinal IR-induced lung injury, but melatonin was more effective in ameliorating the parameters analyzed in this study.

## INTRODUCTION

Intestinal ischemia-reperfusion (iIR) occurs in many clinical situations, including ischemic colitis, acute mesenteric ischemia, septic shock, hemorrhagic or traumatic shock, small bowel transplantation, severe burns, and other conditions that contribute to organ failure with high mortality rates of up to 60% (1-4).

In iIR, the inflammatory response can occur in organs remote from the point of insult (5), resulting in multiple organ dysfunction syndrome (MODS) (6). MODS is a continuous process rather than a single event with incremental degrees of physiological derangements in individual organs. Organ function can be altered by a mild degree of organ dysfunction to completely irreversible organ failure. Lungs are highly susceptible to such injuries and the resulting condition is clinically defined as acute respiratory distress syndrome (ARDS) and is a primary component of MODS. Therefore, it is important to develop therapies and strategies for the prevention and treatment of ARDS after iIR.

N-acetylcysteine (NAC) is an antioxidant, which acts by direct scavenging of reactive oxygen species (ROS), such as hypochlorous acid, hydrogen peroxide, superoxide, and hydroxyl radicals (7). Meanwhile, melatonin (MEL) or N-acetyl-5-methoxytryptamine is synthesized and secreted by the pineal gland, among other organs (8-10).

Melatonin has potent antioxidant and anti-inflammatory effects and acts by upregulating anti-oxidative enzymes and downregulating pro-oxidative enzymes (8-10). Melatonin is a well-known free radical scavenger that can counteract the harmful effects of a variety of molecules (11). Melatonin has protective effects against lung injury induced by ischemia-reperfusion (11). In addition, melatonin has an antioxidant effect in acute lung injury (ALI) caused by sepsis, ischemia-reperfusion, and other situations (11-14), and probably in severe acute respiratory syndrome-coronavirus-2 (SARS-CoV-2) infection.

Taken together, the published reports indicate that NAC and melatonin may interfere with the course of the inflammatory response and exert an important effect on cellular homeostasis. However, there is still some doubt about the role of these agents in the inflammation caused by intestinal ischemic events. In this study, we investigated whether a single dose of melatonin or NAC reduces lung tissue injury following IR of the superior mesenteric artery in rats.

## MATERIALS AND METHODS

All experimental procedures were performed following the established guidelines for the ethical treatment of experimental animals and were approved by the Institutional Animal Care and Use Committee (approval number 6311301018) of the Federal University of São Paulo (UNIFESP- Universidade Federal de São Paulo), in the city of São Paulo, Brazil. Twenty-eight healthy adult male Wistar rats (12-16 weeks old, weighing 230-340 g) were obtained from the Center for the Development of Experimental Models for Biology and Medicine (CEDEME-UNIFESP). The animals were housed in a temperature- and humidity-controlled environment (22±1°C; relative humidity, 50±5%), on a 12/12 h light/dark cycle, with access to a commercial pellet diet and filtered tap water *ad libitum*. We conducted all investigations at the Interdisciplinary Laboratory of Investigation in Surgery at UNIFESP.

The animals were randomly segregated into four experimental groups (n=7 per group) as follows: sham group, in which the animals received an intraperitoneal (i.p.) injection of 0.9% saline solution (2 mL) and were subjected to all surgical procedures, except occlusion of the superior mesenteric artery (not submitted to iIR); SS+iIR group, previously administered (i.p.) with 0.9% saline solution (2 mL) and subjected to 60 min of ischemia, induced by clamping of the mesenteric superior artery, followed by 120 min of reperfusion; NAC+iIR group, pretreated with 20 mg/kg (1.22×10^-4^ mol/kg) of NAC (Fluimucil, Zambon Farmacêutica, SP, Brazil) in 0.9% saline solution (i.p., in a total volume of 2 mL) followed by iIR, as described above; MEL+iIR group, pretreated with 20 mg/kg (8.6x10^-5^ mol/kg) of melatonin in 0.9% saline solution (i.p., in a total volume of 2 mL) followed by iIR, as described above. For treatment of the MEL+iIR group, melatonin (Sigma-Aldrich, MO, USA) was freshly dissolved in absolute ethanol. The resulting solution was diluted with saline to a concentration of 0.2% ethanol in saline. For the SS+iIR and NAC+iIR groups, a volume of ethanol equal to that used for the dissolution of melatonin was added to the saline and NAC solutions, respectively.

The groups were designed as follows:

**Figure:**
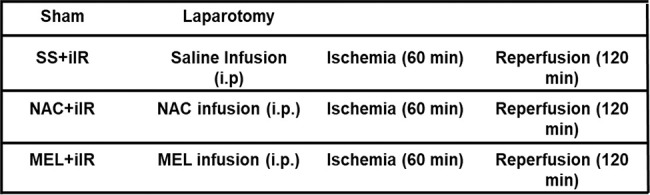


The animals were anesthetized with xylazine hydrochloride (10 mg/kg; Syntec, SP, Brazil) and ketamine hydrochloride (40 mg/kg; Syntec, SP, Brazil). They were placed on a warming pad in supine position and immobilized using adhesive tape. After confirmation of the anesthetic plan and aseptic surgical preparation, 250 IU of heparin (Liquemine, Roche, SP, Brazil) was administered through the left femoral vein to prevent clotting. Subsequently, the femoral vein was used for fentanyl infusion (2 µg/kg/h). We performed a midline laparotomy, and the superior mesenteric artery was identified, dissected, and clamped, for all animals except those in the sham group. The animals received invasive ventilatory support (Model 683 Ventilator, Harvard Apparatus, MA, USA) with a tidal volume (VT) of 5 mL/kg, inspired oxygen fraction (FiO_2_) of 0.21, positive end-expiratory pressure of 3 cmH_2_O, and respiratory rate (RR) of 60-80 breaths/min. The RR for each group was adjusted to achieve normocapnia (arterial carbon dioxide tension, PaCO_2_, 35-45 mmHg). Muscle relaxation was maintained with 0.8 mg/kg (i.p.) pancuronium bromide (Pavulon-Organon, SP, Brazil). The body temperature was monitored using a rectal thermometer.

The surgical site was closed with 2/0 polypropylene sutures to prevent heat loss, while the animals were subjected to the heat pad. After a 60 min period of ischemia, the animals were maintained in supine position and were anesthetized. Then, the clamp was removed and reperfusion was performed for 120 min; additional anesthetic doses were administered as necessary. To prevent corneal desiccation, a bland ophthalmic ointment was administered in the eyes.

Arterial blood samples were obtained from the right carotid after 120 min of reperfusion to evaluate the arterial oxygen tension (PaO2)/fraction of inspired oxygen (FiO2) ratio.

At the end of the experiment, the animals were euthanized with T-61 Euthanasia Solution^®^ (1 mL/100 g; Schering-Plough, SP, Brazil), and necropsy was performed. The lungs were inflated to 25 cmH_2_O for quantitative histological measurements and removed *en bloc*. The left lungs were divided into two parts: the upper part was immersed in a 10% formalin solution, and the lower part was stored at −20°C for subsequent biochemical analysis. The lower part of the left lung tissue was homogenized on ice in 1 mL of 1.15% KCl using a sonicator (Q 700, QSonica, CT, USA) and centrifuged (10,000 rpm at 4°C for 20 min). The supernatant samples were prepared and aliquoted for malondialdehyde (MDA), myeloperoxidase (MPO), superoxide dismutase (SOD), and tumor necrosis factor (TNF)-alpha measurements.

MPO activity levels were studied in the lung tissues and expressed as U/g of protein. MPO activity was analyzed spectrophotometrically, as previously described (15).

MDA levels were determined by thiobarbituric acid reaction, as previously described (16). In the thiobarbituric acid reaction test, malondialdehyde (or malondialdehyde-like substances) reacted with thiobarbituric acid to produce a pink chromogen with peak absorbance at 532 nm on a spectrophotometer. Tissue levels are expressed as nmol/g of tissue.

SOD activity was determined according to a previously described method (17) and assayed as inhibition of the photochemical reduction of nitroblue tetrazolium at 560 nm. SOD activity is expressed as U/g of tissue.

The middle lobe of the right lung was homogenized and centrifuged to obtain the supernatant. The content of TNF-alpha was measured according to the manufacturer’s instructions (Rat TNF-alpha, ELISA kit, RD System, MN, EUA). TNF-α was expressed as pg/mL.

The left lung specimens were inflated and fixed in 10% buffered formalin, processed using standard techniques, and embedded in paraffin. Cross-sectional slices (5-µm thick) were taken from the middle zones of the lungs and mounted on slides. The slides were stained with hematoxylin and eosin, and then examined under light microscopy coupled to a video camera (Axiolab Standard 2.0, AxionCam; Zeiss, Jena, Germany).

Lung injury was scored on a scale of 0 to 3 (0, absent; 1, mild; 2, moderate; 3, severe) (18). Ten fields were randomly selected for evaluation, and the degree of lung injury was expressed by the total score of each animal. All histopathological assessments were performed in a blinded fashion by a pathologist who was blinded to the study.

### Statistical analysis

All results are presented as mean±standard deviation (m±SD). The analytical results were evaluated using the Statistical Package for the Social Sciences, version 17.0 (SPSS Inc., Chicago, IL, USA). One-way analysis of variance (ANOVA; for more than two variables) was used to determine the difference between the groups, and the Tukey test was applied to verify the statistical difference between the groups. Values with *p*<0.05 were considered statistically significant, excluding histological analysis.

## RESULTS

**Figure 1:**
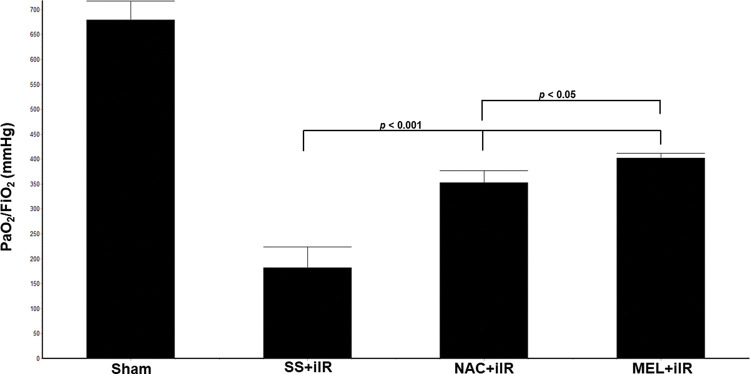
Diagram showing the PaO_2_/FiO_2_ levels of the animal groups. Note that PaO_2_/FiO_2_ levels were significantly reduced before NAC and melatonin administration; the reduction was more evident in the MEL group. Data are shown as the mean±standard deviation of seven rats in each group. Sham: control; SS+iIR: control intestinal ischemia-reperfusion (iIR); NAC+iIR: NAC administration and iIR; MEL+iIR: Melatonin administration and iIR.

All rats survived until the end of the study period. Blood gas analysis (Figure 1) demonstrated a reduction in the oxygen index (PaO_2_/FiO_2_) in the SS+iIR group when compared with the NAC and MEL-pretreated groups (*p*<0.05). Moreover, the MEL+iIR group demonstrated a better response than the NAC+iIR group (*p*<0.05).

**Figure 2:**
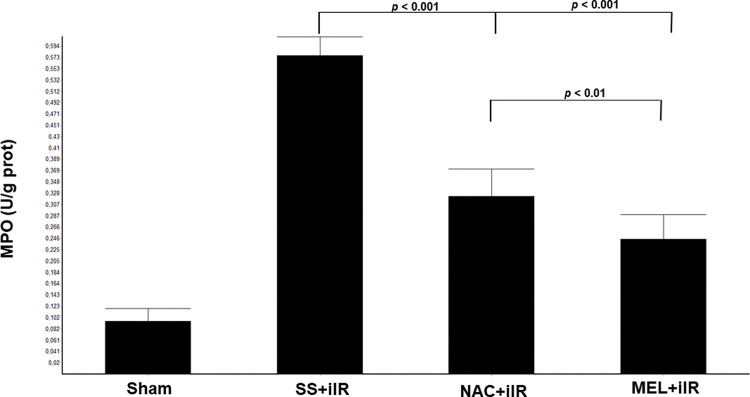
Diagram showing the MPO levels of the animal groups. The MPO levels were significantly reduced before NAC and melatonin administration, the reduction was more evident in the MEL group. Data are shown as the mean±standard deviation of seven rats in each group. Sham: control; SS+iIR: control intestinal ischemia-reperfusion (iIR); NAC+iIR: NAC administration and iIR; MEL+iIR: Melatonin administration and iIR.

MPO activity (Figure 2) was higher in the SS+iIR group (0.57±0.03 U/g of protein) than in the NAC+iIR (0.32±0.05 U/g of protein) and MEL+iIR groups (0.25±0.04 U/g of protein) (*p*<0.001).

**Figure 3:**
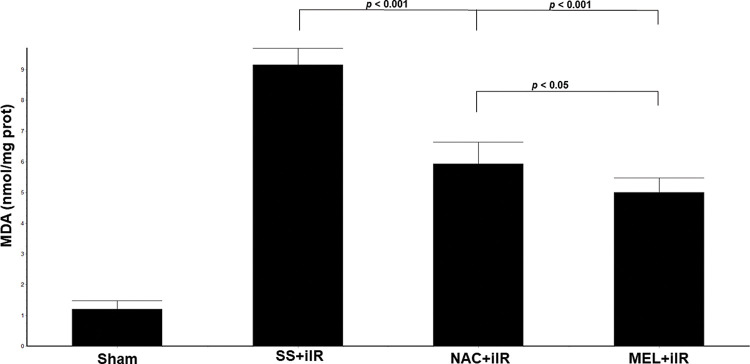
Diagram showing the MDA levels of the animal groups. Note that levels were significantly reduced before NAC and melatonin administration, the reduction was more evident in the MEL group. Data are shown as the mean±standard deviation of seven rats in each group. Sham: control; SS+iIR: control intestinal ischemia-reperfusion (iIR); NAC+iIR: NAC administration and iIR; MEL+iIR: Melatonin administration and iIR.

MDA levels (Figure 3) were higher (*p*<0.001) in the SS+iIR group (9.15±0.54 nmol/mg of protein) than in the NAC+iIR (5.93±0.71 nmol/mg of protein) and MEL+iIR groups (5±0.48 nmol/mg of protein).

**Figure 4:**
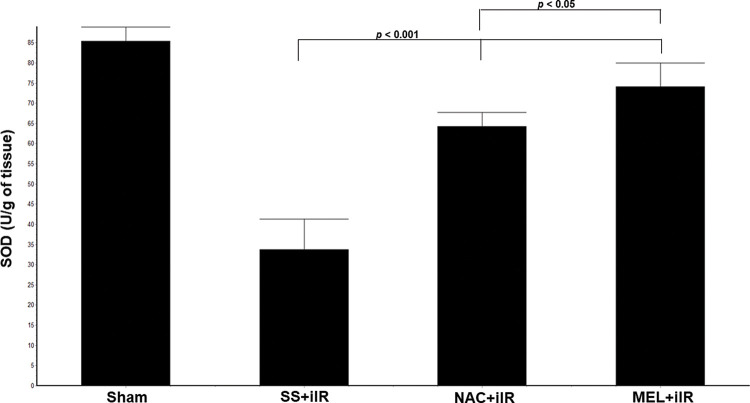
Diagram showing the levels of antioxidant enzymes (superoxide dismutase [SOD]) in the animal groups. The antioxidant enzymes were significantly increased before NAC and melatonin administration, the reduction was more evident in the MEL group. Data are shown as the mean±standard deviation of seven rats in each group. Sham: control; SS+iIR: control intestinal ischemia-reperfusion (iIR); NAC+iIR: NAC administration and iIR; MEL+iIR: Melatonin administration and iIR.

SOD levels (Figure 4) were higher in the NAC+iIR (64±3.5 U/g of tissue) and MEL+iIR (74.2±6 U/g of tissue) groups than in the SS+iIR group (34±8 U/g of tissue; *p*<0.001).

**Figure 5:**
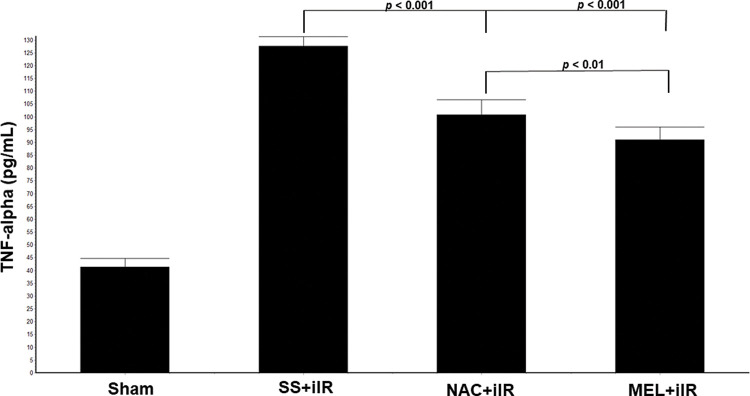
Diagram showing the TNF-alpha levels of the animal groups. Note that TNF-alpha levels were significantly reduced before NAC and melatonin administration, the reduction was more evident in the MEL group. Data are shown as the mean±standard deviation of seven rats in each group. Sham: control; SS+iIR: control intestinal ischemia-reperfusion (iIR); NAC+iIR: NAC administration and iIR; MEL+iIR: Melatonin administration and iIR.

TNF-α levels were higher in the SS+iIR group (128±4 pg/mL) than in the NAC+iIR (101±6 pg/mL) and MEL+iIR (91±5 pg/mL; *p*<0.001) groups. Moreover, TNF-α was lower in the MEL+iIR group than in the NAC+iIR group (*p*<0.01; Figure 5).

**Figure 6:**
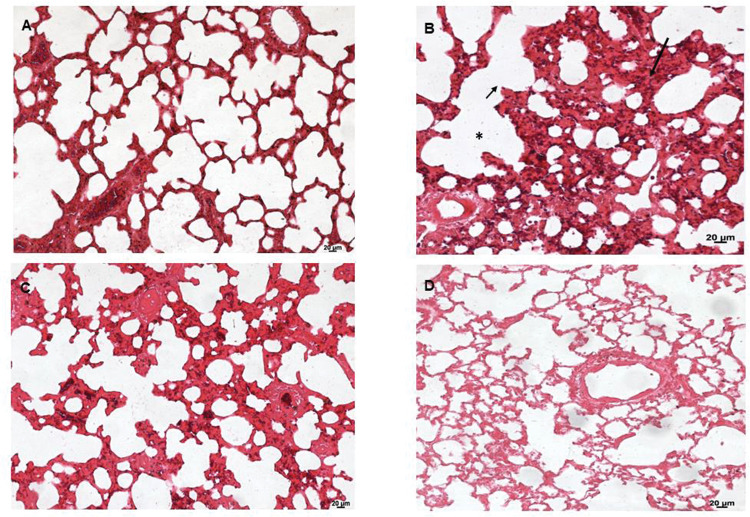
Lung photomicrograph of rats where melatonin and NAC were administered to attenuate lung injury induced by intestinal ischemia-reperfusion (iLR). A (sham): Normal appearance; B (SS+iIR): alveolar edema (*), destruction of the alveolar septum (small arrow), inflammation (long arrow); C (NAC+iIR): moderate edema/inflammation, destruction of the alveolar septum, and congestion; D (MEL+iIR): mild to moderate congestion, reduced inflammation, and destruction of the alveolar septum. Hemoatoxylin eosin 200X.

Lung tissues were analyzed under a light microscope. As shown in Figure 6, the sham group (A) exhibited normal lung tissue structures. In contrast, severe damage to the lung area was observed in the SS+iIR group (B). However, MEL (D) and NAC (C) pretreatment ameliorated the iLR-induced lung injury, but the MEL+iIR group exhibited greater improvement when compared with the NAC+iIR group. Histopathological examination showed a normal appearance of the lung parenchyma in the sham group (Figure 6A). Furthermore, the SS+iIR group demonstrated a higher score (2.86±0.38; *p*<0.001) when compared with those of the NAC+iIR (2.28±0.49) and MEL+iIR (1.85±0.37) groups.

## DISCUSSION

In the present study, we demonstrated that NAC or melatonin pretreatment can prevent iIR-induced lung injury in rats. Injury induced by iIR is related to a wide range of clinical conditions, including neonatal necrotizing enterocolitis, midgut volvulus, obstructive acute abdomen, incarcerated hernia, sepsis, and hemorrhagic shock (19,20). Lung damage occurs following transient arterial occlusion. Ischemic damage results from a decrease in blood flow to an organ. When restoring blood flow, more pronounced damage occurs, called reperfusion injury. IR injury involves increased generation of oxygen radicals. Free radicals have already been shown to play a significant role in the etiology of remote lung injury in animal models. It has often been shown that lung injury generally occurs after skeletal muscle ischemia because of the release of free radicals from reperfused tissues (11).

A wide variety of animal models have been used to identify the mechanisms and potential treatments of iIR, such as clamping of the intestinal mesenteric artery. In this study, we applied a widely used model by clamping the superior mesenteric artery to explore the effects of melatonin and NAC pretreatment on ALI induced by iIR. Okutan et al., using rats pretreated with 20 mg/kg of melatonin and subjected to lung injury by infrarenal aortic occlusion for 30 min and reperfusion for 12h, demonstrated a significant reduction in MPO activity and MDA levels (21). Our data demonstrated that melatonin treatment reduced oxidative stress, as reflected by the attenuation of MPO and MDA levels.

MPO plays a fundamental role in the production of potential oxidants by neutrophils. Neutrophils are the main source of oxygen-free radicals. A previous study clearly demonstrated that skeletal muscle IR led to remote lung inflammation, which was characterized by increased MPO activity and significant neutrophil infiltration into the lungs. Nitric oxide level is a significant determinant of lung injury processes caused by lower extremity iIR (11,22-24).

A previous study showed that clamping the superior mesenteric artery for 45 min led to the recruitment of neutrophils (22-24). Several studies have confirmed the accumulation of PMNs in both local and remote vascular tissues by iIR (25,26), and the accumulation of PMNs has been demonstrated by an increase in MPO and MDA. The results of the present study showed that melatonin or NAC pretreatment reduced MDA and MPO levels.

Lung injury after iIR may activate PMNs through a process that requires the involvement of ROS (27). Our study corroborates this data as a reduction of MPO and MDA and increased SOD levels were observed in the pretreated rats. This investigation also extends the information by demonstrating better effects in the group pretreated with melatonin, which showed a reduction in MDA and increase in SOD levels, indicating that MEL could provide protection against lung injury in our model.

Melatonin and its metabolites have antioxidant properties *in vitro*, both in cells and body fluids. Furthermore, melatonin plays an important role in activating antioxidant defenses and protects cells from oxidative loads and apoptosis induced by mitochondrial DNA damage (28-31). Our study confirmed a decrease in the oxidative response with increased SOD levels after melatonin pretreatment.

Proinflammatory cytokines are reduced in rats pretreated with melatonin (32-35) and NAC (36) in other conditions. Our study shows a better effect of melatonin than NAC in the reduction of TNF-alpha or proinflammatory mediators.

NAC has toxic side effects, such as nausea, vomiting, diarrhea, and gastrointestinal irritation (36,37). However, no side effects of melatonin at any dose have been described (38-41).

Intestinal IR occurs in several clinical conditions and often leads to damage to other organs, including the lungs, causing multiple organ failure. The results are devastating to the patient and can even lead to death.

This study showed that the intestine has local inflammatory effects before and after reperfusion. The products generated locally do not remain contained. In this way, we understand the intestine as the “engine” of inflammation and because it has access to other compartments, it allows inflammatory mediators to amplify the systemic response.

Our study has some limitations. First, we did not investigate the role of NAC and melatonin in lung injury induced by iIR through inflammatory pathways. The exact inflammatory and oxidative pathways need to be elucidated in the future. Second, our research was performed using an animal model, and thus, further clinical research is required. Third, arterial blood tests were performed only at the end of reperfusion, and evaluation over an extended period of time is necessary. In addition, other serum biochemical markers have not yet been evaluated.

## CONCLUSION

In conclusion, our study shows that pretreatment with NAC or melatonin has the potential to protect against injuries to remote organs, such as the lungs. Moreover, pretreatment with melatonin improved gas exchange, attenuated inflammatory cytokines and oxidative stress, and reduced lung injury severity in our mesenteric IR rat model.

## AUTHOR CONTRIBUTIONS

Leite AA and Camargo CR performed the NAC and melatonin treatment, technical procedures, and acquisition of the data. Brandão JCM, Sakae TM and Marinho M were responsible for the interpretation of the data and critical revision of the manuscript. Reiter RJ contributed to the data analysis and critical revision of the manuscript. Oliveira-Junior IS was responsible for the design and intellectual and scientific content of the study and manuscript writing. All of the authors have approved the final version of the manuscript for publication.
